# Adherence to TB Treatment in Ethiopia: Why Do Patients Default?

**DOI:** 10.1371/journal.pmed.0040165

**Published:** 2007-04-24

**Authors:** Hundie Tesfaye

Tuberculosis as a disease has been present in humans since antiquity, with the earliest unambiguous detection of Mycobacterium tuberculosis in the remains of bison dated 17,000 years before the present [1]. About 90% of those infected with TB have asymptomatic (latent) infection. One in ten latent infections may progress to active disease which, if left untreated, kills more than half of its victims [2]. In 2004, 14.6 million people had active TB and there were 8.9 million new cases and 1.7 million deaths [3], mostly in developing countries, including Ethiopia.

Shargie and Lindtjørn explain the physical lack of access to the treatment centre as the main cause of failure to adherence to therapy in 20% of patients [4]. Only 52 patients indicated they lived over 10 km from the treatment centre. It is not clear whether all the 52 are among those who failed to complete the doses. It was of interest whether all 64 patients from the urban area (near to the treatment centre) completed the treatment. Nevertheless, it might be worthwhile to compare the rate of completion of treatment of the 64 urban dwellers with those 52 individuals to support the idea that physical access to the treatment centre is the main factor for treatment adherence.

Almost half of the patients enrolled in the study earned 0–99 Ethiopian birr (approximately 0–10 US dollars) per month. However, it is not clear from the report whether all 74 (91%) who failed to complete the treatment belonged to those with low monthly income. Furthermore, the fate of those earning more than 200 birr (approximately US$20) was not clear in terms of treatment completion. The authors reported that income had no influence on the outcomes in terms of treatment completion, despite their inclusion of cost of transport among the factors which led to incompletion of therapy.

Unfortunately the study did not evaluate the data of family numbers and its influence on treatment adherence in relation to family income. Larger family size with inappropriate income may contribute to malnutrition, leading to drug intolerance and general malaise and finally to loss of motivation to continue the treatment course.

The authors stated there was no influence of HIV status on the defaulting of treatment, despite the known fact that more than half of the cohort studied did not volunteer for the HIV test.

Drug-resistant strains of TB have emerged and are spreading dangerously. In 2000–2004, 20% of cases were resistant to standard treatments, and 2% were also resistant to second-line drugs [5]. In spite of only one treatment failure here, extensively drug-resistant TB may be a possible challenge in Ethiopia. Whether Ethiopia succeeds in the Stop TB Partnership's Global Plan to Stop Tuberculosis, which aims to save 14 million lives between 2006 and 2015 (see http://www.stoptb.org/globalplan), depends on the effectiveness of the national program, infrastructure development, peace, and good governance with sustainable development assistance from donors directed to improving the life condition of the Ethiopian people, so that the population is self-sufficient and confident enough to overcome burning issues like TB.

In conclusion, the study confirms that TB drug delivery, without implementation of anti-poverty programs and more access to public health facilities, is ineffective.
